# Spontaneous Resolution of Tension Pneumocephalus Following Blunt Head Trauma

**DOI:** 10.7759/cureus.5027

**Published:** 2019-06-28

**Authors:** Joshua Tsau, Rohan Mangal, Latha Ganti, Kendra Amico

**Affiliations:** 1 Emergency Medicine, University of Central Florida College of Medicine/Hospital Corporation of America Graduate Medical Education Consortium, Orlando, USA; 2 Emergency Medicine, John Hopkins University, Baltimore, USA; 3 Emergency Medicine, Envision Physician Services, Orlando, USA; 4 Emergency Medicine, University of Central Florida College of Medicine, Orlando, USA

**Keywords:** tension pneumocephalus, noninvasive

## Abstract

The authors present a case of tension pneumocephalus that occurred secondary to closed head injury and review the etiology and management of this relatively rare entity. This case was managed without invasive neurosurgical intervention, also somewhat rare for this condition.

## Introduction

Pneumocephalus is a collection of air or gas within the intracranial cavity and is a common finding following traumatic skull fractures and neurosurgical procedures [1]. Tension pneumocephalus develops when intracranial air accumulates under pressure due to a one-way valve effect similar to tension pneumothorax [2]. Common mechanisms include head or facial trauma, basilar skull tumors, a neurosurgical or otorhinolaryngological procedure, and rarely barotrauma from scuba diving or even positive pressure ventilation [2-3]. The clinical presentation of tension pneumocephalus varies from a simple headache to seizures, lethargy, and altered mental status [3-4]. Tension pneumocephalus may lead to subsequent compression of the frontal hemispheres, along with a widening of the interhemispheric space, giving a peaked appearance due to intact bridging veins [2]. This phenomenon, seen on computed tomography (CT) results, in the characteristic “Mount Fuji sign” and is considered a neurosurgical emergency [3]. Early recognition by radiologists and emergency physicians, with prompt neurosurgical consult and consideration of antibiotics to prevent meningitis, is imperative.

## Case presentation

We report the case of a 62-year-old male with a history of alcoholism who presented to the emergency department by ambulance, complaining of a frontal headache after a ground-level fall from standing with associated head trauma. His family reported that the patient had been weak and dizzy in the preceding four days with recurrent falls. However, this particular event resulted in the patient being briefly unresponsive, which prompted them to call 911 (emergency medical services). Physical examination was notable for a right supraorbital laceration. Neurological examination revealed intact cranial nerves and no focal motor or sensory deficits. He was afebrile, with normal blood pressure, pulse, and respiration. His Glasgow coma scale (GCS) score was 14/15 (E4 V4 M6).

**Figure 1:**
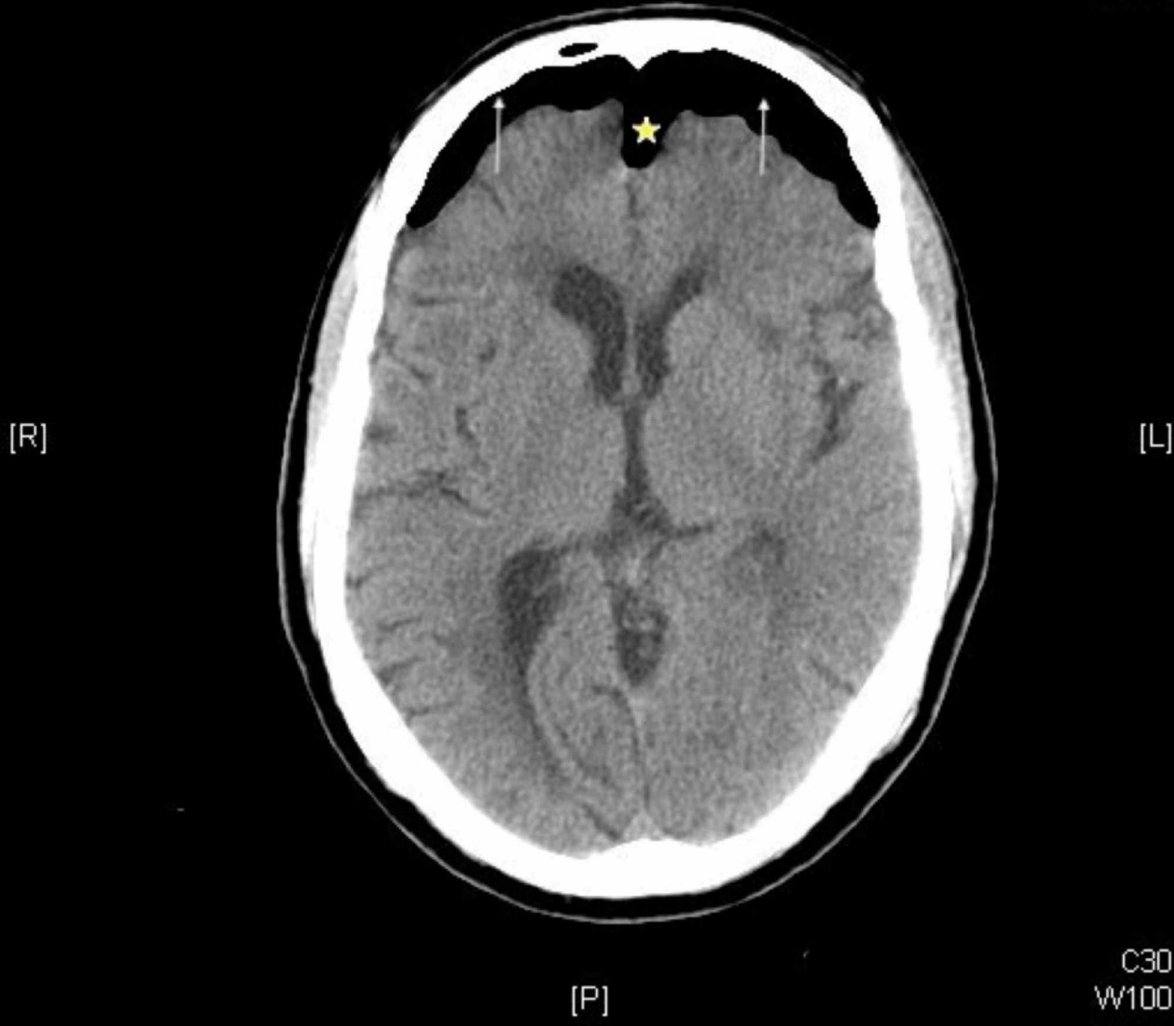
Noncontrast brain CT demonstrates subdural air (arrows). There is separation of the brain parenchyma from the falx cerebri, classically referred to as the Mount Fuji sign (star).

**Figure 2:**
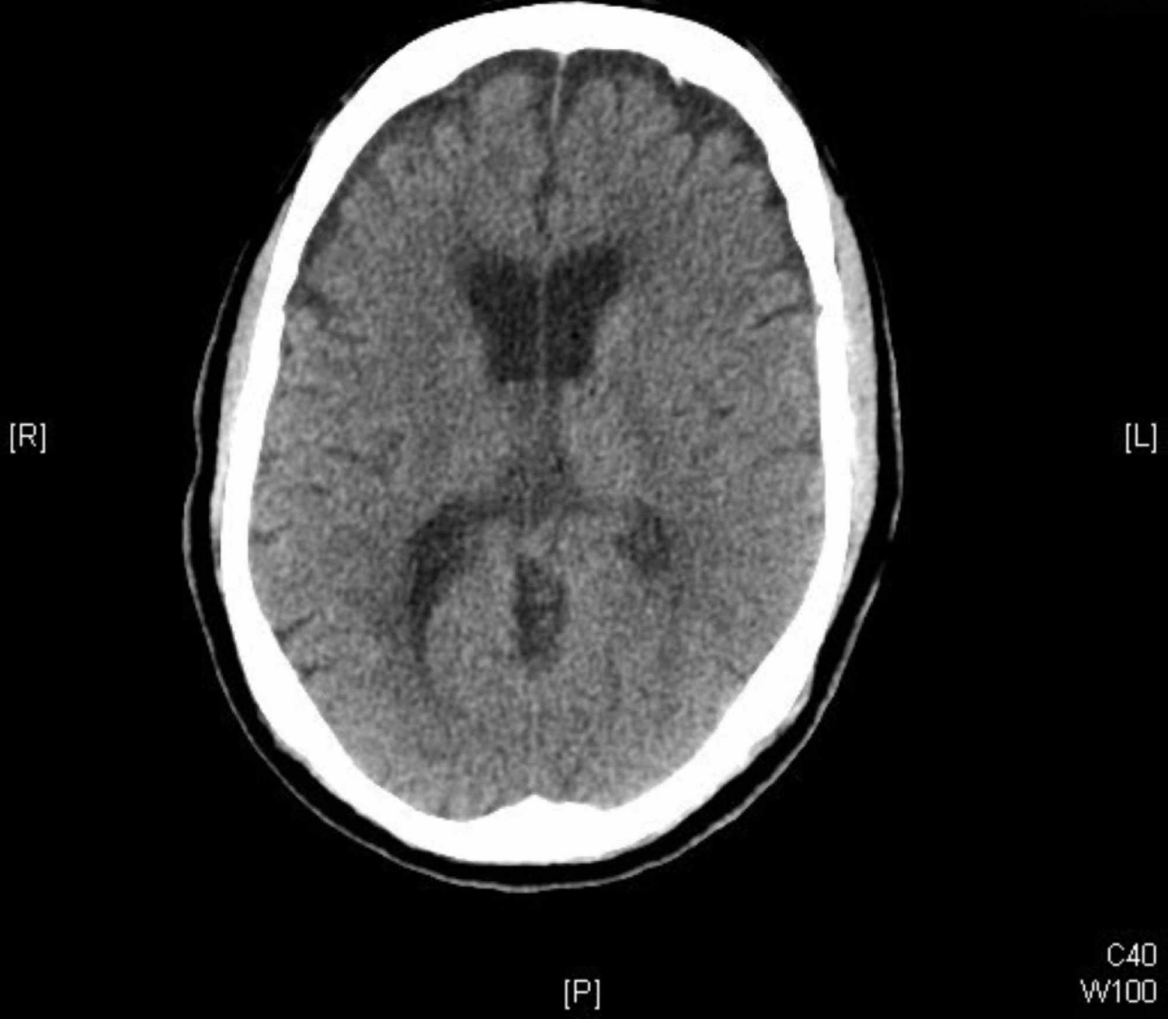
Noncontrast brain CT demonstrates resolution of the pneumocephalus.

The noncontrast head CT revealed no acute fractures but showed subdural air with separation of the frontal lobes by subdural air-findings concerning for tension pneumocephalus (Figure 1). Neurosurgery was subsequently consulted. The patient was admitted and managed conservatively with the elevation of the head of the bed, analgesia, close neurologic monitoring, and instructions to restrict nose blowing or coughing. The supraorbital laceration was repaired with topical skin adhesive. By the third hospital day, the patient’s headache and dizziness resolved, and the repeat CT demonstrated complete resolution of the pneumocephalus (Figure 2). The patient was subsequently discharged home.

## Discussion

There are several mechanisms by which pneumocephalus develops. With a skull fracture or neurosurgical procedure, direct inoculation of the intracranial space with air is expected. Dural injuries can lead to tension pneumocephalus when air enters through the defect while a plug of brain parenchyma or dural membrane prevents that same air from exiting, creating a ball-valve effect [4-5]. Another theory has been termed the "inverted soda bottle effect": excessive cerebrospinal fluid (CSF) loss results in negative intracranial pressure; extracranial air then enters replacing the CSF allowing pressure to equalize between the two spaces [6]. However, in the absence of calvarial disruption, there are a few hypothesized mechanisms for how air enters the intracranial space. Case reports exist of the intravenous injection of air causing pneumocephalus [7]. Lastly, the production of gas may develop in situ due to infection by a gas-forming organism such as Clostridium perfringens or Enterobacter cloacae [8-9].

Conservative treatment of tension pneumocephalus involves semi-recumbent positioning with head-of-bed elevation to 30-45 degrees, avoidance of Valsalva maneuvers, pain control, prevention of hyperthermia, and osmotic diuretics [10]. With conservative management, reabsorption of subdural air occurs in 85% of cases after two to three weeks [11]. The administration of supplemental oxygen through a nonrebreather mask significantly increases the absorption rate of postcraniotomy pneumocephalus as compared with breathing room air or nasal cannula [12]. Hyperbaric oxygen, in some studies, has been shown to decrease the rates of meningitis as well as decrease the length of hospital stay. If conservative and supplemental measures do not lead to a resolution of the pneumocephalus, operative interventions can be pursued. More aggressive options include drilling of burr holes to create an open as opposed to a ball-and-valve system, needle aspiration, or closure of a known dural or bony defect. In this case, conservative measures were sufficient to produce a rapid resolution of the pneumocephalus.

## Conclusions

What we learned from this case is that tension pneumocephalus is a relatively rare neurosurgical emergency that can sometimes be managed conservatively. The presenting symptoms can be nonspecific, as with our patient. Thus, maintaining a high index of suspicion for this pathology is very important. Once suspected, the diagnosis is made relatively easily on noncontrast CT.
